# Prevalence and Factors Associated with Impairment in Intrinsic Capacity among Community-Dwelling Older Adults: An Observational Study from South India

**DOI:** 10.1155/2023/4386415

**Published:** 2023-04-22

**Authors:** Abhijith Rarajam Rao, Mujtaba Waris, Mamta Saini, Meenal Thakral, Karan Hegde, Manjusha Bhagwasia, Prabha Adikari

**Affiliations:** ^1^Geriatric Oncology, Department of Medical Oncology, Tata Memorial Centre, Parel, Mumbai, India; ^2^Department of Geriatric Medicine, All India Institute of Medical Sciences, New Delhi, India; ^3^Department of Geriatric Medicine, Yenepoya Medical College, Mangalore, Karnataka, India

## Abstract

**Background:**

Intrinsic capacity (IC) is conceptualized by World Health Organization (WHO) with a focus on healthy aging. Identifying impairment could help in making a person-centred plan for the care of older adults.

**Objectives:**

Establish the prevalence of IC among community-dwelling older adults age >60, the prevalence of impairment in each domain, and identify factors associated with an impairment in IC.

**Methods:**

This cross-sectional observational study in the community setting included 1000 older adults aged 60 years and above in two-year study period. The 6 domains of IC including cognition, locomotor capacity, psychological, vitality, hearing, and vision were derived from the comprehensive geriatric assessment. The IC composite score was calculated based on these domains, and a higher IC score indicated greater IC.

**Results:**

During the study period, 1000 older adults, with the median age of 66.5 (IQR-63-73) were included, and 629 (62.9%) were women. Only in 157 (15.7%) community-dwelling older adults, all 6 domains were intact. Impairment in one, two, and three domains was seen in 442 (42.2%), 305 (30.5%), and 91 (9.1%), respectively. The most prevalent impaired domain was locomotor (593, 59.3%), followed by vision (441, 44.1%), hearing (193, 19.3%), cognition (106, 10.6%), mood (38, 3.8%), and vitality (37, 3.7%). The factors associated with lower IC included increasing age (*β*-coefficient −0.01, 95% CI: −0.02 to −0.01, *p* value = 0.002), impaired activities of daily living (*β*-coefficient −0.13, 95% CI: −0.49 to −0.18, *p* value <0.001), and chronic neurologic illness (*β*-coefficient −0.10, 95% CI: −0.77 to −0.18, *p* value = 0.001).

**Conclusions:**

In conclusion, we found that impairment in IC was frequent in community-dwelling older adults, and it is associated with age, presence of chronic neurologic illness, and declining functionality. The adoption of IC should be seen as an opportunity to disseminate geriatric care in our healthcare systems which lack the necessary attention to the needs of older persons.

## 1. Introduction

Advancing age of the global population is associated with increasing disability, which is a major challenge for the healthcare system [1]. The World Health Organization (WHO) has conceptualized the health and healthcare of older adults around the concept of healthy ageing. Healthy ageing is defined as the process of developing and maintaining the functional ability that enables well-being in older age. In addition, it depends upon an individual's intrinsic capacity (IC), environment, and the interactions between the two [2]. Intrinsic capacity is the composite of all the physical and mental capacities individuals can draw upon at any point in their life [3]. It includes all the processes that help individuals to maintain independence.

The five different domains of IC proposed to operationalise the concept include cognition, mood, locomotion, vitality, and sensory domains (hearing and vision) [4]. These domains influence each other and, in turn, are influenced by environmental factors impacting the older person's functional ability. A continuous measure of IC and its trajectory in conjunction with the surrounding environment will help to track the functionality not only at the individual level but at the community level as well. This may, in turn, facilitate interventions to preserve IC and necessary changes in the environment, both at the individual and community level, to enhance and maintain the functional ability of older persons.

Multiple studies from India have reported frailty in hospital and community settings [5–8]. Few studies from India have recently reported on IC as well [9, 10]. This study aimed to establish the prevalence of impaired IC among community-dwelling older adults aged ≥60 and the prevalence of impairment in each domain. We also intended to identify factors associated with an impairment in IC.

## 2. Materials and Methods

The current study is a post hoc analysis of a cross-sectional study carried out in the community setting in a coastal city of South India. Four localities attached to a tertiary Care Medical college hospital in Mangalore, a coastal district in Karnataka, South India, were chosen for the main study. From each locality, 250 older adults were taken, giving a total sample size of 1000. The study duration was two-year. The study was initiated after obtaining ethical clearance from Institutional Ethics Committee. Community-dwelling older adults aged 60 years and above who consented to the evaluation were included, and people who were bed-bound with severe acute illness were excluded. A trained healthcare staff conducted a face-to-face interview to fill out a detailed questionnaire from the WHO Age friendly Primary Health Centres (PHC) toolkit consisting of seven parts. The domains of IC, including cognition, locomotion, psychological, vision, hearing, and vitality, were derived from the CGA.

The details included age, gender, socioeconomic status according to the modified Kuppuswamy scale, marital status, and use of substances such as tobacco smoking and alcohol consumption. Height and weight were measured and body mass index (BMI) was measured as weight in kg divided by height squared in metres. Functional status was evaluated using basic activities of daily living (ADL) and instrumental activities of daily living (IADL). Geriatric syndromes included falls, urinary incontinence, constipation, and insomnia. Comorbidities, such as previous diagnosis of hypertension, diabetes, chronic respiratory disease, cardiovascular diseases, and chronic neurologic illness were assessed. Multimorbidity has been defined as presence of two or more comorbidities.

### 2.1. Domains of Intrinsic Capacity and Scoring

The domain of cognition was assessed using Hindi-Mental Status Examination (HMSE) [11, 12]. The locomotor capacity was evaluated using the timed-up and go-test [13, 14]. The psychological capacity was assessed using the Geriatric depression scale-15 (GDS 15) [15]. The sensory domain included hearing assessment using a whisper test and vision using a screening question “Do you have any difficulty in seeing a car from a long distance or reading or difficulty in doing any of your daily activities because of your eyesight?.” The reply was considered positive if the subject replied as yes and negative if the answer was no. Vitality was assessed using body mass index (BMI).

A HMSE <24 was considered as impairment in cognitive domain. A TUG >13 seconds was used to identify impairment in locomotor domain. A GDS score ≥ 5 was considered abnormal. An inability to hear all three words in both the ears in a whisper test and a “yes” response for vision screen was abnormal. For vitality, a BMI < 18.5 kg/m^2^, which is the cut-off score for undernutrition in Asia Pacific population [16], was used to identify impairment in vitality. Total maximum IC was 6 (one point for each domain). An impairment in even a single domain was considered as impaired IC.

### 2.2. Statistical Analysis

Statistical analysis was performed using STATA version 14 (StataCorp. 2015. Stata Statistical Software: Release 14. College Station, TX: StataCorp LP.). We did not do a priori sample size calculation. Continuous variables are described as mean, standard deviation or median, and interquartile range, and categorical variables are expressed as frequencies and percentages. The association between categorical variables and IC score was analysed using Wilcoxon rank-sum test. For variables with more than two categories, Kruskal–Wallis test was used. If a significant main effect was found on the Kruskal–Wallis test, then a post-hoc analysis was performed using the Mann-Whitney *U* test to explain the significant main effect using Bonferroni correction. Complex linear regression was used to find the association of various factors with IC after adjustment for age and gender. The results are presented as beta-coefficient and 95% confidence interval (CI). To find association between individual domains and factors, logistic regression was used. The results are represented as odds ratio (OR) with 95% CI. A *p* value of <0.05 was considered statistically significant.

## 3. Results

**Figure 1:**
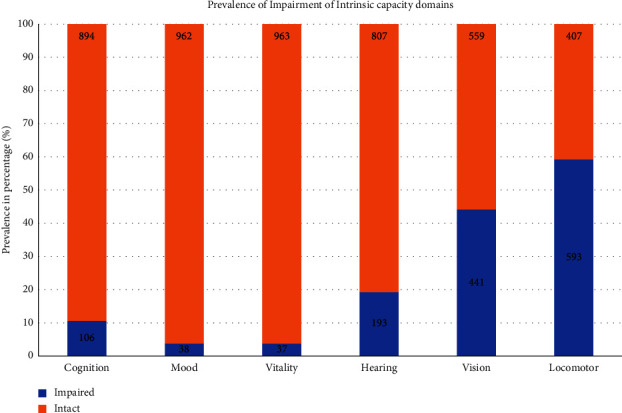
Prevalence of impairment of individual intrinsic capacity domains.

A total of 1000 community-dwelling older adults were included in this study, and among them, 157 (15.7%) had intact IC (Figure 1). The median age (IQR) of the study population was 66.5 (63–73) years, and 629 (62.9%) were female.

Table 1 describes the baseline characteristics, comorbidities, and geriatric syndromes. Only 30 and 59 participants were current smokers and consumed alcohol, respectively. The ADL was impaired in 179 (17.9%) of the participants. More than two-thirds of the population (689, 70.2%) had a BMI more than 22.9 kg/m^2^.

Among the factors, age category (60–74 and ≥ 75 years) (*p* value = 0.011), ADL category (intact and impaired) (*p* value <0.001), and chronic neurologic illness (*p* value = 0.002) were significantly associated with IC score. Gender, socioeconomic status, marital status, substance abuse, geriatric syndromes, and comorbidities (other than chronic neurologic illness) were not associated.

Further linear regression (Table 2) revealed that age >75 years was negatively correlating with IC score (*β*-coefficient: −0.17, 95% CI: −0.32 to −0.03, *p* value: 0.015). Impaired ADL (*β*-coefficient: −0.13, 95% CI, −0.49 to −0.18, *p* value <0.001) and chronic neurologic illness (*β*-coefficient −0.10, 95% CI: −0.77 to-0.18, *p* value = 0.001) were also negatively associated with IC score even after adjusting for age and gender. In addition, IADL scores correlated positively with IC score (*β*-coefficient 0.06, 95% CI, 0.01 to 0.9, *p* value = 0.018).

Figure 1 describes the prevalence of impaired IC domains. Among the six domains of IC, locomotor impairment was the most prevalent (593, 59.3%), followed by the vision (441, 44.1%). At the same time, the vitality was the least impaired domain (39, 3.8%). In this community setting, 157 (15.7%) of subjects had no impairment in any domain of IC, whereas most of the participants (422, 42.2%) had impairment in one domain. While 3 (0.3%) participants had impairment in five domains (Table 3).

Table 4 reports the results of logistic regression, determining the factors associated with different domains of IC.

## 4. Discussion

We report the prevalence of impairment in the IC domains and the factors associated with IC score of Indian community-dwelling older adults. The main findings of this study are (a) 15.7% have all IC domains intact, (b) IC declines with ageing, (c) increasing age, presence of chronic neurological illness, impaired ADL, and lower IADL score are associated with impaired IC, and (d) most impaired domains are locomotion and vision.

The prevalence of impaired IC in our population was 84.3%, which was much higher than a community-based cross-sectional study from China, which reported a prevalence of 39.9% [17]. This difference could be due to the different tools used to measure various domains of IC as well as the difference in the definition of impaired IC. Among the Chinese older adults, it was reported that locomotion was the most impaired domain (17.8%), followed by sensory (14.2%). We found that locomotion (59.3%) and vision (44.1%) are most commonly impaired domains, and the proportion was much higher. Also, the previous study was carried out at multiple sites across the country, and our study was carried out in a single community setting. These affected domains could be targets for intervention to preserve the autonomy of older adults, as impaired IC is associated with an increased risk of disability and falls, fractures, frailty, immobility and incident dependence, and death [17–19].

Of interest here is how the impairment in the domains of IC varies. A study which included data from Latin America, Indian, and China reported that there was considerable variation in the prevalence of individual capacities [18]. The prevalence of maintained vitality varied between 66% and 98.7%, vision capacity and hearing capacity was between 59.8% and 93.5% and 76.9% and 96.8%, respectively. Cognitive capacity varied from 33.2% to 90.3%, and psychological capacity was 62.0% to 98.5%. Interestingly the prevalence of full capacity also varied from 12.0% to 62.8%. These findings highlight the importance of establishing region-specific implementation of IC in the care needs of older adults.

We found that increasing age is a risk factor for impairment in IC, which mirrors previous studies [17, 18]. Similar to these studies, our study demonstrates an association between the presence of chronic neurologic disease with impaired IC. Morbidity (dementia, depression, and stroke) and disability were more common with declining IC [17]. However, we did not find any association between gender, education, marital status, substance abuse, and socioeconomic status. We also found that 84.3% of older adults had an impairment in one or more domains of IC, which is similar to previous studies (69–89%) [20–22].

This study reports on the status of IC in Indian community-dwelling people and the need for urgent intervention to prevent the consequences of impaired IC. Previous studies note that a decline in IC is independently associated with an increased risk of functional decline, falls, and mortality. This will lead to an increased burden on the healthcare system and a detrimental effect on individuals [18, 21, 23]. To identify these vulnerable older adults, the WHO proposed “Integrated Care for Older People (ICOPE)” could be implemented at the primary health care level. This will be essential for optimising the IC of older adults, helping them maintain their functional ability, and strengthening the concept of healthy ageing.

We acknowledge that the single community setting and the cross-sectional study design are major limitations. Due to the cross-sectional nature, we cannot establish a causal relation. Further longitudinal studies are urgently required to establish the relationship between the decline in IC and poor health-related outcomes, including mortality. We used a total composite score instead of a more appropriate weighted score. A study is required to establish a tool to measure the IC using culturally appropriate tools and the cut-off for older Indian adults.

## 5. Conclusion

In conclusion, we found that decline in IC was frequent in community-dwelling older adults, and it is associated with age, presence of comorbidities, and declining functionality. Our research indicates that promoting IC to delay the decline in the functional capacity of older adults should focus on managing comorbidities and improving vision and locomotor domains. The adoption of IC should be seen as an opportunity to disseminate geriatric care in our healthcare systems which lack the necessary attention to the needs of older persons.

## Figures and Tables

**Table 1:** Baseline characteristics of the study population (*n* = 1000).

Variable	*N* (1000)	Intrinsic capacity score (out of 10)	*p* value
Age (median (IQR))			
Age			
<75 years	787	5 (4-5)	0.011
≥75 years	213	5 (4-5)	
Gender			
Male	371	5 (4-5)	0.231
Female	629	5 (4-5)	
Socioeconomic status			
Upper	205	5 (4-5)	0.060
Upper middle	408	5 (4-5)	
Lower middle	234	5 (4-5)	
Upper lower	88	5 (4-5)	
Marital status			
Married	963	5 (4-5)	0.144
Widow/widower	4	3.5 (3–4.5)	
Unmarried	33	5 (4-5)	
Substance use			
Smoking	30	5 (4-5)	0.985
Alcohol	59	4 (4-5)	0.129
ADL			
Impaired	179	4 (4-5)	<0.001
Intact	821	5 (4-5)	
BMI category (kg/m^2^)			
<18.5	37	4 (3-4)	<0.001
18.5–22.9	256	5 (4-5)	
23–24.9	206	5 (4-5)	
25–29.9	340	5 (4-5)	
>30	143	5 (4-5)	
Geriatric syndromes			
Falls			
Yes	62	5 (4-5)	0.525
No	919	5 (4-5)	
Urinary incontinence			
Yes	79	5 (4-5)	0.766
No	908	5 (4-5)	
Constipation			
Yes	34	5 (4-5)	0.170
No	952	5 (4-5)	
Insomnia			
Yes	46	5 (4–6)	0.064
No	937	5 (4-5)	
Comorbidities			
Multimorbidity			
Yes	164	5 (4-5)	0.071
No	835	5 (4-5)	
Hypertension			
Yes	293	5 (4-5)	0.178
No	706	5 (4-5)	
Diabetes			
Yes	168	5 (4-5)	0.612
No	831	5 (4-5)	
Chronic respiratory illness			
Yes	93	5 (4-5)	0.393
No	906	5 (4-5)	
CVD			
Yes	41	5 (4-5)	0.689
No	958	5 (4-5)	
Chronic neurologic illness			
Yes	43	4 (3–5)	0.002
No	955	5 (4-5)	

^*∗*^BMI: post-hoc analysis: significant different between <18.5 and 18.5–22.9 BMI (*p* − 0.008), <18.5 and 23-24.9 (*p* − 0.008), and <18.5 and 25-29.9 (*p* − 0.009). BMI: body mass index, ADL: activity of daily living, and CVD: cardiovascular disease.

**Table 2:** Association of factors with impaired intrinsic capacity.

Variables	Beta-coefficient	95% confidence interval	*p* value
Age	−0.01	−0.02 to −0.01	0.002
Age category			
60–75	0 (reference)		
≥75	−0.17	−0.32 to −0.03	0.015
Gender			
Male	0 (reference)		
Female	0.06	−0.06 to 0.18	0.349
BMI	0.01	−0.01 to 0.12	0.742
ADL			
Score	0.18	0.14 to 0.28	<0.001
Intact	0 (reference)		
Impaired	−0.13	−0.49 to −0.18	<0.001
IADL score	0.06	0.01 to 0.9	0.018
Geriatric syndromes			
Falls	−0.02	−0.34 to 0.15	0.450
Urinary incontinence	−0.01	−0.25 to 0.18	0.764
Constipation	0.03	−0.17 to 0.48	0.351
Insomnia	0.31	−0.03 to 0.59	0.065
Comorbidities			
Multimorbidity	−0.05	−0.30 to 0.01	0.083
Hypertension	−0.03	−0.19 to 0.07	0.366
Diabetes	−0.01	−0.17 to 0.14	0.839
Chronic respiratory illness	−0.02	−0.28 to 0.12	0.449
CVD	−0.01	−0.33 to 0.26	0.794
Chronic neurologic illness	−0.10	−0.77 to −0.18	0.001

BMI: body mass index, IADL: instrumental activities of daily living, and CVD: cardiovascular disease.

**Table 3:** Prevalence of number of domains impaired.

No. of impaired domains	Prevalence (percentage)
5	3 (0.3%)
4	22 (2.2%)
3	91 (9.1%)
2	305 (30.5%)
1	422 (42.2%)
0	157 (15.7%)

**Table 4:** Factors associated with domains of intrinsic capacity.

Variables	Cognition	Hearing	Vision	Vitality	Locomotion	Mood
	OR (95% CI)	OR (95% CI)	OR (95% CI)	OR (95% CI)	OR (95% CI)	OR (95% CI)
Age						
Continuous	1.00 (0.97–1.03)	1.07^*∗∗*^ (1.05–1.09)	0.98 (0.96–1.00)	1.04^*∗*^ (1.00–1.09)	1.01 (0.99–1.03)	1.00 (0.86–1.05)
<75 years	Ref	Ref	Ref	Ref	Ref	Ref
≥75 years	1.16 (0.72–1.87)	2.26^*∗∗*^ (1.59–3.20)	0.71^*∗*^ (0.52–0.97)	1.19 (0.55–2.57)	1.57^*∗∗*^ (1.14–2.16)	0.83 (0.36–1.91)
Gender						
Male	Ref	Ref	Ref	Ref	Ref	Ref
Female	0.85 (0.56–1.28)	0.72^*∗*^ (0.52–0.98)	1.01 (0.78–1.31)	1.41 (0.69–2.89)	0.98 (0.75–1.27)	1.01 (0.51–1.98)
SEC						
Upper	Ref	Ref	Ref	Ref	Ref	Ref
Upper middle	0.93 (0.54–1.59)	1.24 (0.80–1.91)	1.70^*∗∗*^ (1.21–2.39)	0.89 (0.38–2.5)	1.23 (0.87–1.73)	0.54 (0.22–1.29)
Lower middle	0.82 (0.44–1.52)	1.09 (0.67–1.78)	0.90 (0.61–1.33)	0.97 (0.39–2.44)	0.90 (0.62–1.31)	0.60 (0.22–1.61)
Upper lower	0.79 (0.34–1.84)	0.92 (0.47–1.81)	0.94 (0.56–1.57)	0.25 (0.03–2.00)	0.74 (0.45–1.22)	1.68 (0.62–4.58)
Marital status						
Married	Ref	Ref	Ref	Ref	Ref	Ref
Unmarried	0.25 (0.03–1.89)	0.92 (0.38–2.28)	0.81 (0.41–1.68)	1	0.92 (0.46–1.88)	1
Smoking						
No	Ref	Ref	Ref	Ref	Ref	Ref
Yes	0.94 (0.28–3.14)	2.50^*∗*^ (1.17–5.35)	0.73 (0.34–1.55)	0.89 (0.12–6.71)	0.78 (0.37–1.61)	1
Alcohol						
No	Ref	Ref	Ref	Ref	Ref	Ref
Yes	0.95 (0.40–2.28)	2.09^*∗*^ (1.18–3.69)	1.00 (0.59–1.70)	0.45 (0.06–3.19)	1.35 (0.78–2.36)	0.43 (0.06–3.19)
ADL score						
Continuous	0.69^*∗∗*^ (0.59–0.83)	0.81^*∗*^ (0.69–0.96)	0.97 (0.83–1.13)	1.66 (0.69–3.93)	0.46^*∗∗*^ (0.33–0.64)	0.73^*∗*^ (0.57–0.94)
ADL category						
Intact	Ref	Ref	Ref	Ref	Ref	Ref
Impaired	1.90^*∗*^ (1.18–3.06)	1.34 (0.89–1.99)	1.08 (0.77–1.51)	0.58 (0.20–1.67)	2.44^*∗∗*^ (1.67–3.56)	1.60 (0.74–3.46)
IADL score						
Continuous	0.94 (0.82–1.08)	0.75^*∗∗*^ (0.67–0.83)	1.01 (0.93–1.09)	1.03 (0.82–1.29)	0.99 (0.91–1.08)	1.07 (0.85–1.35)
Hypertension						
No	Ref	Ref	Ref	Ref	Ref	Ref
Yes	0.68 (0.42–1.09)	1.21 (0.87–1.70)	1.11 (0.85–1.47)	0.77 (0.36–1.64)	1.26 (0.95–1.66)	0.98 (0.48–2.00)
Diabetes						
No	Ref	Ref	Ref	Ref	Ref	Ref
Yes	0.54 (0.28–1.04)	0.80 (0.52–1.25)	1.18 (0.84–1.65)	1.16 (0.50–2.69)	1.33 (0.94–1.88)	0.57 (0.20–1.63)
Chronic respiratory illness						
No	Ref	Ref	Ref	Ref	Ref	Ref
Yes	0.89 (0.43–1.83)	0.93 (0.53–1.61)	0.074 (0.48–1.15)	1.19 (0.41–3.43)	1.67 (1.05–2.65)	2.75^*∗*^ (1.22–6.18)
CVD						
No	Ref	Ref	Ref	Ref	Ref	Ref
Yes	0.65 (0.19–2.16)	0.57 (0.22–1.47)	0.99 (0.53–1.85)	1	2.98^*∗∗*^ (1.34–6.44)	1
Chronic neurologic illness						
No	Ref	Ref	Ref	Ref	Ref	Ref
Yes	1.99 (0.90–4.43)	1.11 (0.52–2.35)	1.21 (0.66–2.24)	1.28 (0.29–5.51)	3.70^*∗∗*^ (1.63–8.41)	3.68^*∗*^ (1.36–9.94)
Multimorbidity						
No	Ref	Ref	Ref	Ref	Ref	Ref
Yes	0.82 (0.46–1.46)	0.92 (0.59–1.42)	0.96 (0.68–1.34)	1.42 (0.64–3.18)	2.02^*∗∗*^ (1.39–2.93)	1.38 (0.62–3.06)

^*∗*^ *p* value <0.05. ^*∗∗*^*p* value <0.005. SEC: socioeconomic status, ADL: activity of daily living, IADL: instrumental activities of daily living, and CVD: cardiovascular disease.

## Data Availability

The de-identified individual data are available on reasonable request from Dr Prabha Adikari by email The data will be available starting from the date of publication onwards.
